# High Isolation Single-Pole Four-Throw RF MEMS Switch Based on Series-Shunt Configuration

**DOI:** 10.1155/2014/605894

**Published:** 2014-02-23

**Authors:** Tejinder Singh, Navjot Khaira

**Affiliations:** Department of Electronics and Communication Engineering, Lovely Professional University, Phagwara 144 402, India

## Abstract

This paper presents a novel design of single-pole four-throw (SP4T) RF-MEMS switch employing both capacitive and ohmic switches. It is designed on high-resistivity silicon substrate and has a compact area of 1.06 mm^2^. The series or ohmic switches have been designed to provide low insertion loss with good ohmic contact. The pull-in voltage for ohmic switches is calculated to be 7.19 V. Shunt or capacitive switches have been used in each port to improve the isolation for higher frequencies. The proposed SP4T switch provides excellent RF performances with isolation better than 70.64 dB and insertion loss less than 0.72 dB for X-band between the input port and each output port.

## 1. Introduction

Microelectromechanical Systems (MEMS) with their extremely small dimensions and full integration into the Radio Frequency (RF) front ends of various telecommunication devices have emerged as the most promising technology over the past few decades. There is an increasing demand of devices having extremely high performance at low power consumption and compact size in various satellite, defence, and communication systems. The RF MEMS switches allow for high isolation in open-circuit state, low insertion loss, and high linearity [1], which is very important in RF subsystems. Different types of MEMS single-pole single-throw (SPST) switches have been proposed [2–5] and have provided excellent RF performances. But in modern communication systems, switches are mostly used in the form of switch matrices for signal routing or reconfiguration where a high number of inputs are connected with the corresponding outputs. Previous attempts of development and use of MEMS multithrow RF switches [6–9] rather than the SPST switches as the fundamental blocks have simplified the integration problem of large size switch matrices due to outstanding RF performances, good extendibility of throws, and a symmetrical transmission behavior.

A high isolation, low loss single-pole four-throw (SP4T) switch with a total area of 1.06 mm^2^ using both metal-contact and capacitive RF MEMS switches is presented in this paper. The signal line is divided into four ports, and the routing of the signal to one of the four output ports is decided by the state of the switches in the respective port and the others. In order to provide high isolation at higher frequencies, a shunt capacitive switch is provided after each ohmic contact switch. The RF performances of the SP4T switch with and without the use of shunt capacitive switches are compared. The symmetrical design of the input port and four output ports allows for its easy extension to more numbers of throws. Such a symmetrical design is also favorable for its application in various communication systems.

## 2. Design Methodology

**Figure 1:**
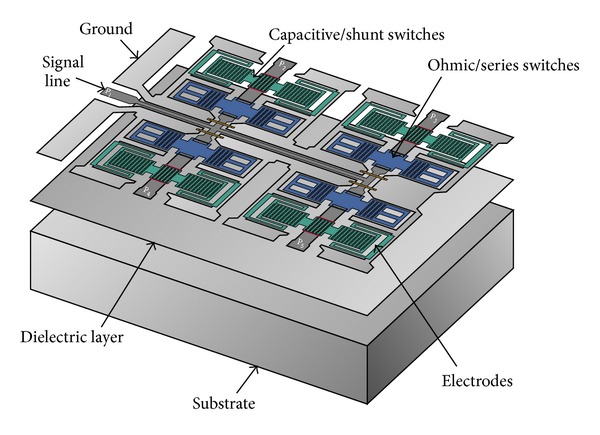
Separated layers of designed SP4T switch. For high isolation operation each output port (*P*_2_ to *P*_5_) has ohmic and capacitive switch membranes in series-shunt configuration. Switch is designed on a dielectric layer over a substrate.

**Figure 2:**
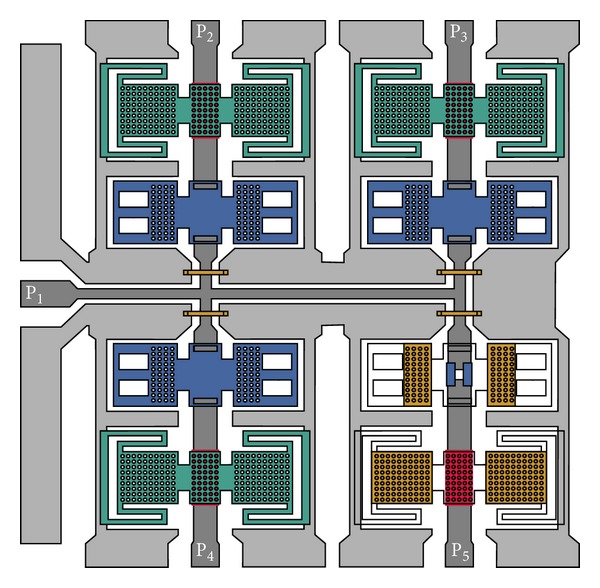
Top view of SP4T switch is shown. At the output ends capacitive switch membranes are placed in shunt configuration (connected to ground planes). The ohmic switches has two-piece metal contact to connect the signal line. In the lower right region, membranes are made transparent to demonstrate electrodes, dielectric layer, and contact points beneath.

The proposed SP4T MEMS switch is comprised of a coplanar waveguide and eight MEMS switches on a silicon substrate [10] as shown in Figure 1. The input signal line is divided into four ports and each one has a series MEMS contact switch and a shunt/capacitive MEMS switch as shown in Figure 2.

*P*_1_ is the input port and *P*_2_ to *P*_5_ are output ports. To route the input signal to one of the output ports, the ohmic contact switch in that port is electrostatically actuated to ON state (down state) and the capacitive switch is kept in up state while the capacitive switches in the other ports are turned OFF (down state) to offer high isolation. The metal contact switch provides the contact between the spliced signal line and the signal is routed to the corresponding output port. Bridges are used to connect the various ground planes.

### 2.1. Coplanar Waveguide Structure

**Figure 3:**
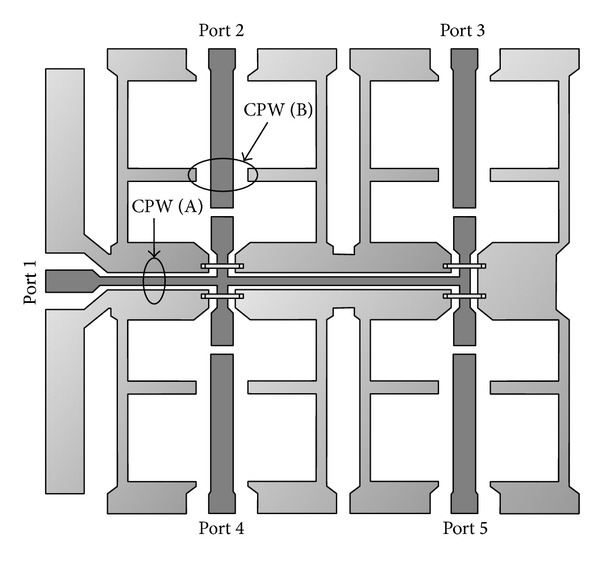
Coplanar waveguide structure is shown with two different types of CPW, and specifications are taken for better propagation of signal. CPW (A) dimensions are for the signal from input source to output ports. CPW (B) is for all the four output regions.

The basic structure of coplanar waveguide (CPW) comprises of a symmetric arrangement with signal strip width *W* and equal longitudinal gap, *G*. Finite-ground coplanar waveguide (FG-CPW) is used in this design where the ground is not shared by two or more lines and hence results in a lower coupling of the adjacent lines. Coplanar waveguides are preferred as they allow easy surface mounting of the devices (here switches), and the reduced dispersion and radiation loss.  Figure 3 shows the two different dimensions of the coplanar waveguide, namely, CPW (A) and CPW (B) as used in this design.

The dimensions for CPW (A) and CPW (B) are specified in Table 1. If *C* is the capacitance per unit length of the line, and *C*_0_ is the capacitance per unit length of line in the absence of dielectric layer, then the effective permittivity for finite ground CPW is given by [11]

(1) $$\begin{array}{c} \varepsilon_{\text{eff}}=\frac{C}{C_{0}}=1+\frac{1}{2}\left(\varepsilon_{r}-1\right)\frac{K\left(k\right)}{K\left(k^{\prime }\right)}\times \frac{K\left(k_{s}^{\prime }\right)}{K\left(k_{s}\right)}, \end{array}$$

where *K* is the complete elliptical integral of the first kind and the values of *k* and *k*′ are decided by the geometry of the line as follows:

(2) $$\begin{array}{c} k=\frac{c}{b}\sqrt{\frac{b^{2}-a^{2}}{c^{2}-a^{2}}},\\ k^{\prime }=\sqrt{1-k^{2}}=\frac{a}{b}\sqrt{\frac{c^{2}-b^{2}}{c^{2}-a^{2}}}. \end{array}$$

The thickness of the substrate (500 *μ*m) ≫ thickness of CPW line (1 *μ*m); hence for infinitely thick substrate *k*_*s*_ = *k*. The expression for effective permittivity reduces to

(3) $$\begin{array}{c} \varepsilon_{\text{eff}}=\frac{1}{2}\left(\varepsilon_{r}+1\right), \end{array}$$

where *ε*_*r*_ is the relative permittivity of the substrate. Further the phase velocity is given by

(4) $$\begin{array}{c} V_{p}h=\frac{c^{\prime }}{\sqrt{\varepsilon_{\text{eff}}}}, \end{array}$$

where *c*′ is the speed of light in free space. The characteristic impedance *Z*_0_ is given as

(5) $$\begin{array}{c} Z_{0}=\frac{30\pi }{\sqrt{\varepsilon_{\text{eff}}}}\times \frac{K\left(k\right)}{K\left(k^{\prime }\right)}. \end{array}$$

The CPW (A) with characteristic impedance value of 50 Ω satisfies the demand of RF input matching and also the size considerations. The dimensions of SP4T switch are given in Table 1.

The dielectric layer used in this proposed SP4T design is of Hafnium Dioxide (HfO_2_) with dielectric constant, *k* = 25.

## 3. Ohmic Contact Switches

### 3.1. Membrane Designing

**Figure 4:**
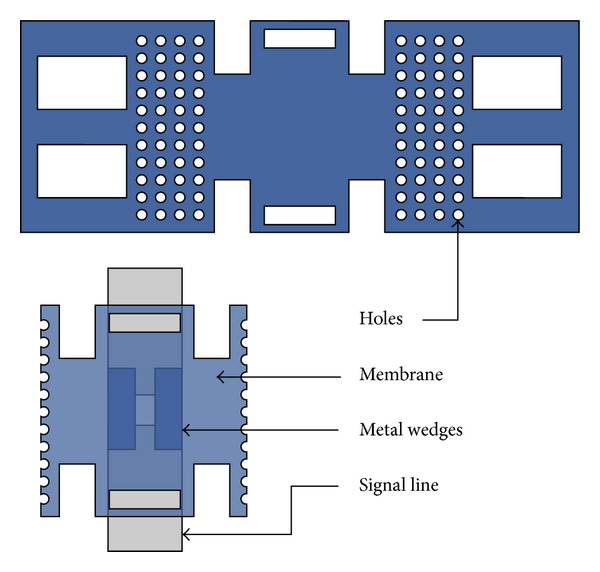
Series/ohmic switch membrane is demonstrated, and the bottom image shows the membrane spliced from middle and placed on signal line. At the bottom of membrane the etal wedges or contact pads to connect the signal line are also demonstrated.

Ohmic contact switches are used in this proposed design in conjunction with shunt switches.  Figure 4 shows a closer view of metal-contact RF MEMS switch used in the proposed SP4T switch. These switches are placed in series with the single line and have two pull-down electrodes for actuation. Holes are provided in the membrane to lower the spring constant so as the actuation voltage.

The conduction of the signal between the two ends of the RF line depends on the position of the switch membrane above the signal line. Applying bias voltage to the two pull-down electrodes actuates the switch; thus, the membrane is pulled down and two gold contact wedges of 1 *μ*m thickness establish the contact between the broken signal lines. When the bias voltage is removed, the membrane restores back to the OFF state; hence, it acts as a mechanical relay. The dimensions of various parts of the metal-contact switch are listed in Table 2. It is further discussed in Section 5; the isolation characteristic depends upon the gap between the broken signal lines as well. But if we approach to increase this gap, then overall dimensions of the switching part also increase. Hence, to improve the isolation, we have used capacitive RF MEMS switch in each arm after the metal-contact switch.

### 3.2. Spring Constant and Pull-In Voltage Analysis

The spring constant (*k*) for the metal contact RF MEMS switch with the membrane fixed at the extreme ends is modeled in two parts. The first part, *k*′, is due to the rigidity of the movable beam and the other part, *k*′′, is due to biaxial residual stress in the beam as a result of the fabrication process [1]:

(6) $$\begin{array}{c} k=k^{\prime }+k^{\prime \prime }. \end{array}$$

**Figure 5:**
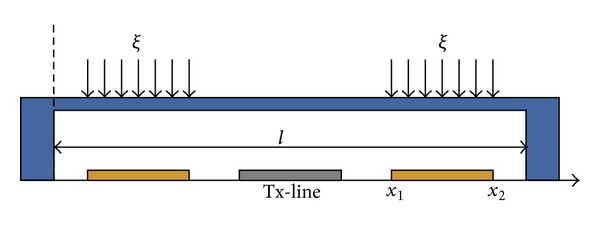
The force model is applicable on both membranes due to the twin electrodes used to actuate the membrane, *l* is the length of membrane, and *ξ* is the force applied in downward direction from *x*_1_ to *x*_2_ per electrode.

The deflection of the beam at the center is used to determine the spring constant for MEMS switches. In this case, the load is distributed as shown in Figure 5; hence, the deflection is given as

(7) $$\begin{array}{c} y=\frac{2}{EI}\int_{x_{1}}^{x_{2}}\frac{\xi }{48}\left(l^{3}-6l^{2}a+9la^{2}-4a^{3}\right)‍, \end{array}$$

where *ξ* is the load per unit length, so that the total load is

(8) $$\begin{array}{c} P=\xi \left(2\left(x_{1}-x_{2}\right)\right). \end{array}$$

The moment of inertia, *I*, is given as

(9) $$\begin{array}{c} I=\frac{wt^{3}}{12}, \end{array}$$

where *w* is the width and *t* is the thickness of the membrane. The part of the spring constant *k*′ can be computed using

(10) $$\begin{array}{c} k^{\prime }=-\frac{P}{y}=-\frac{\xi \left(2\left(x_{1}-x_{2}\right)\right)}{y}. \end{array}$$

Biaxial residual stress for this proposed design is given by the expression as

(11) $$\begin{array}{c} k^{\prime \prime }=8\sigma \left(1-v\right)w\left(\frac{t}{l}\right)\frac{1}{3-2\left(x/l\right)}. \end{array}$$

By using the approximated value of the spring constant *k*, the pull-in voltage can be calculated numerically as

(12) $$\begin{array}{c} V_{\text{pull-in}}=\sqrt{\frac{8kg_{0}^{3}}{27\varepsilon_{0}A}}, \end{array}$$

where *g*_0_ is the gap height and *A* is the area of the electrode. After putting the value listed in Table 2 in (12), the pull-in voltage is calculated to be 7.19 V.

## 4. Capacitive Switches

**Figure 6:**
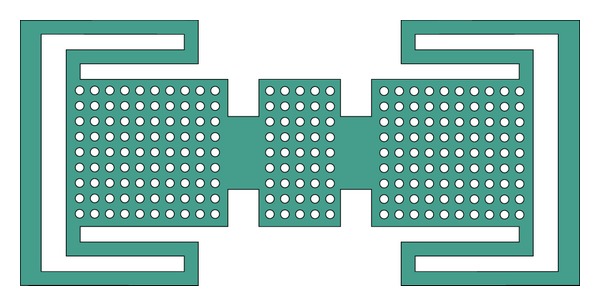
Capacitive/shunt membrane for SP4T switch. Dielectric layer is used beneath the middle part of membrane that covers the signal line to vary the capacitance. Both ends are connected to ground planes of CPW.

Shunt capacitive switches are used before each output port to offer high isolation [12] in the proposed SP4T switch design. The capacitive MEMS switch used in each arm is shown in Figure 6.

It has two wide pull-down electrode areas and has a meander based design so as to lower the driving voltage. When DC actuation voltage is applied to the pull-down electrodes, the electrostatic force causes the membrane to collapse on the dielectric layer above the signal line, which largely increases the bridge capacitance. The capacitance couples the signal line to the ground and acts as a short circuit. When the bias voltage is removed, the membrane returns back to its original rest state due to the restoring forces in the membrane. The dimensions of the capacitive switch are shown in Table 3.

### 4.1. Hafnium Dioxide as a Dielectric Material

The conventional dielectric materials like SiO_2_ and Si_3_N_4_ used in MEMS switches show dielectric charging phenomenon due to charge trapping in the layer [13]. This can be overcome by the use of high conductivity materials in which charges flow away more easily and for this reason we have used high-*k* dielectric material HfO_2_ with dielectric constant *k* = 25.

### 4.2. Perforation in Membrane

Holes with diameter 3 *μ*m are provided in the membrane to attain faster switching speeds as they reduce the air damping effect underneath the bridge.

Holes also ease the removal of sacrificial layer during the fabrication process. Moreover, holes also release some of the residual stress in the beam and reduce the mass of the beam resulting in a higher mechanical resonant frequency [1].

## 5. Electromagnetic Circuit Modeling

**Figure 7:**
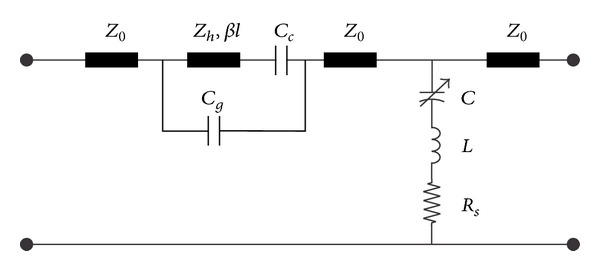
Equivalent circuit model of single port of proposed SP4T switch. Due to series-shunt configuration, the *CLR* model of series switch is combined with shunt as given. From left *Z*_0_ to middle *Z*_0_ component, the circuit is for ohmic switches and then till the rightmost component *Z*_0_, the circuit is applicable for capacitive switches with variable *C*.

As previously discussed, each arm of the SP4T design consists of a series metal-contact and a shunt capacitive switch.  Figure 7 shows the equivalent circuit model of one arm of SP4T switch, when the series switch is put to OFF state.

The off-state capacitance of the series metal-contact MEMS switch is composed of two factors: *C*_*g*_ and *C*_*c*_. *C*_*g*_ is the signal-line coupling capacitance in the OFF state due to the gap between the broken signal lines. *C*_*c*_ is the contact capacitance between the *t*-line and the contact metal of the movable membrane. As in this case we have two contact areas, the OFF-state capacitance of the series switch, *C*_*s*_ is given as

(13) $$\begin{array}{c} C_{s}=\frac{C_{c}}{2}+C_{g}. \end{array}$$

For the capacitive MEMS switch placed in shunt between the *t*-line and the ground, the shunt impedance is given by

(14) $$\begin{array}{c} Z_{s}=R_{s}+j\omega L+\frac{1}{j\omega C}. \end{array}$$

The value of capacitance depends on the position of the membrane above the signal line. When the membrane is in the up state the capacitance is given as

(15) $$\begin{array}{c} C_{u}=\frac{\varepsilon_{0}wW}{g+\left(t_{d}/\varepsilon_{r}\right)}+C_{f}, \end{array}$$

where *C*_*f*_ is the fringing field capacitance. The capacitance of the shunt switch in down state is given as

(16) $$\begin{array}{c} C_{d}=\frac{\varepsilon_{0}\varepsilon_{r}wW}{t_{d}}. \end{array}$$

The *LC* resonant frequency of the switch *f*_0_ is given as

(17) $$\begin{array}{c} f_{0}=\frac{1}{2\pi \sqrt{LC}}. \end{array}$$

The switch behaves as a capacitor below the *LC* resonant frequency and as an inductor above the resonant frequency. At resonance, the switch behaves as a resistance in the circuit.

## 6. RF Performance Analysis

### 6.1. Isolation Performance

**Figure 8:**
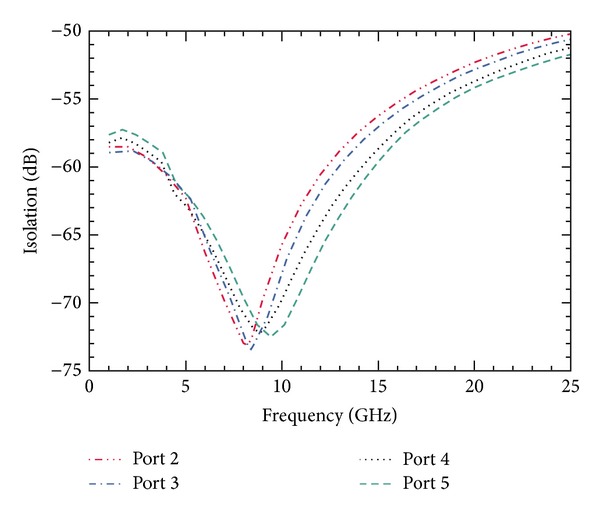
Simulation of isolation performance of SP4T switch for a frequency sweep of 1–25 GHz results in excellent isolation of *≈*72.5 dB at 8 GHz.

The simulation to obtain the isolation between the input port and the output ports is done using commercially available EM modeler. The SP4T switch is enclosed in a vacuum box, so that no environmental factors can affect the results. The simulated results for isolation between the input port and different output ports over a range of frequencies are shown in Figure 8. The well-known dip in the curves is due to the *LC* resonance in capacitive switches.

### 6.2. Comparison of Isolation

**Figure 9:**
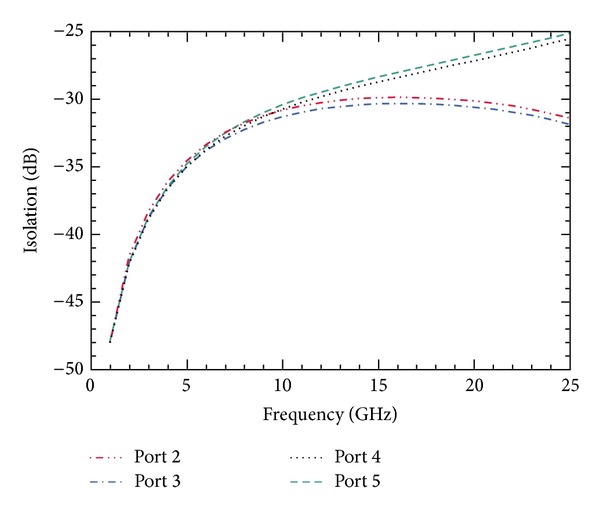
Isolation plot with only ohmic/series switch. The maximum isolation is 33.7 dB at 8 GHz and more than 40 dB for operation below 2 GHz.

Shunt capacitive switches have been applied in each arm to improve the isolation.  Figure 9 shows the simulated values of isolation in the OFF state between input port and each output port when the shunt switches are not applied.

**Figure 10:**
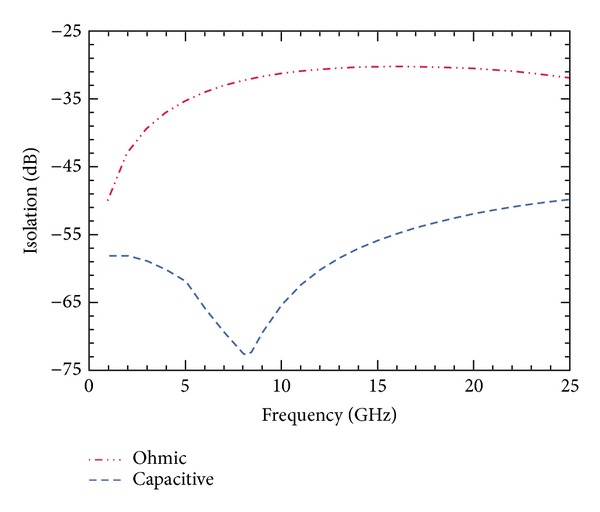
Comparison of the isolation performance of designed SP4T switch and its impact on the inclusion of capacitive switches is shown. The isolation increases more than double.

Figure 10 shows the comparison between the isolation curves with and without the use of shunt capacitive switches. Hence, the improvement in isolation achieved is clearly observed. Whereas the switch offered an isolation of 33.7 dB between port *P*_1_ and *P*_2_ at 8 GHz without the use of shunt switches, the isolation improved to *≈*72.5 dB by the inclusion of shunt switches in each port.

### 6.3. Insertion Loss and Return Loss

**Figure 11:**
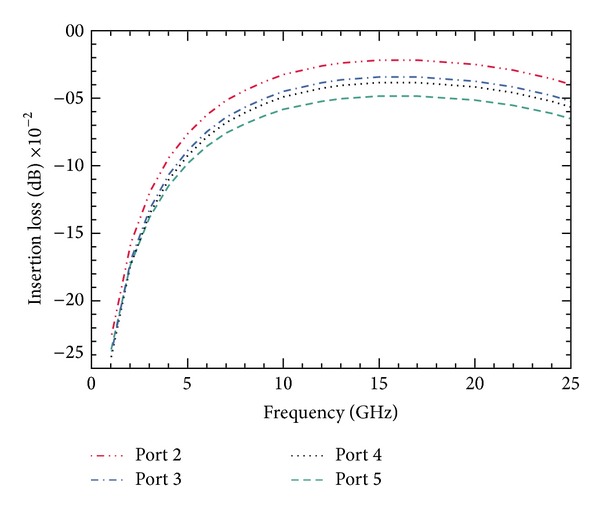
Simulation of insertion loss parameter. The simulation is carried out for all the ports results in insertion loss *≈*0.06 dB at 8 GHz frequency. Due to the series-shunt configuration, the insertion loss is consistent for frequencies above 6 GHz and performance is low for frequencies below 6 GHz.

The insertion loss offered by the proposed switch when the signal is routed between the input port and one of the output ports over a range of frequencies is shown in Figure 11. The measured insertion losses between the input port and each output port (*P*_2_, *P*_3_, *P*_4_, and *P*_5_) at 8 GHz are 0.041, 0.052, 0.057, and 0.071 dB, respectively.

**Figure 12:**
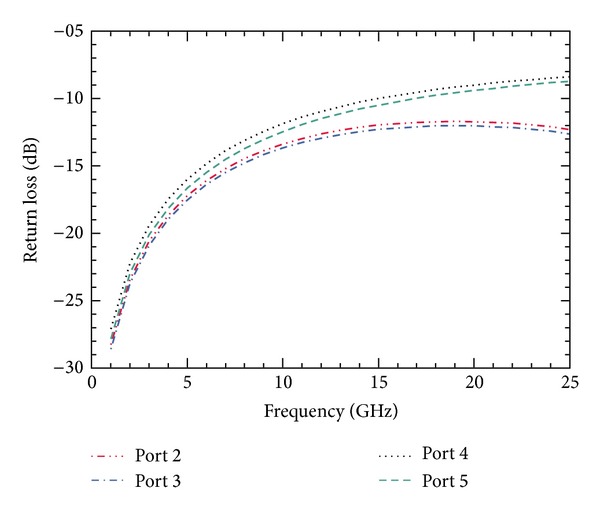
Return loss plot of designed switch in ON state for the frequency sweep of 1–25 GHz results in return loss of *≈*14 dB at 8 GHz; the graph points out that below 8 GHz, the return loss increases, so as the performance of the RF MEMS switch.

The simulated values of return loss of the SP4T switch between the input port and each output port (*P*_2_, *P*_3_, *P*_4_, and *P*_5_) at 8 GHz in the ON-state mode are 8.07, 9.45, 9.37, and 7.62 dB, respectively. The graph displaying the return loss over the range of frequencies is shown in Figure 12. From the results it is clear that the designed SP4T switch provides very high isolation by taking benefit from series-shunt configuration.

## 7. Conclusion

The design and modeling of a RF MEMS SP4T switch based on series-shunt switches has been presented. The proposed switch results in excellent RF performance with isolation better than 72 dB at 8 GHz. Such high isolation has been achieved by the use of shunt capacitive switches in each output port. The switch has a very compact size with a symmetrical arrangement of the switches and the ports, which makes it suitable for application in many communication systems, space systems, and satellites.

## Figures and Tables

**Table 1:** Switch specifications.

Component	Length *μ*m	Width *μ*m	Depth *μ*m	Material
Substrate	1000	1060	500	Silicon
Substrate dielectric	1000	1060	0.5	HfO_2_
CPW (G S G) (A)	14	20	14	Gold
CPW (G S G) (B)	28	40	28	Gold

**Table 2:** Dimensions of ohmic RF MEMS switches.

Parameter	Dimensions	Material
Contact switch	316 × 120 *μ*m^2^	Gold
Pull-down electrode × 2	50 × 120 *μ*m^2^	Gold
Gap height, *g*_0_	2 *μ*m	—
Hole diameter	3 *μ*m	—
Contact metal × 2	15 × 80 *μ*m^2^	Gold
Anchors	10 × 120 *μ*m^2^	Gold

**Table 3:** Dimensions of capacitive RF MEMS switches.

Parameter	Dimensions	Material
Contact switch	378 × 180 μm^2^	Gold
Pull-down electrode × 2	110 × 100 μm^2^	Gold
Gap height, *g*_0_	3 μm	—
Hole diameter	3 μm	—
Dielectric area	40 × 100 μm^2^	HfO_2_
Dielectric thickness	0.15 μm	Gold
